# Supplementary material for: Prevalence of anemia and its associated factors among under-five children living in Arba Minch Health and Demographic Surveillance System Sites (HDSS), Southern Ethiopia
Source: PLOS Glob Public Health. 2024 Nov 5;4(11):e0003830. doi: 10.1371/journal.pgph.0003830 (PMC11537392; doi:10.1371/journal.pgph.0003830)
Supplement: S1 Text — (DOCX) [file pgph.0003830.s001.docx]

**S1 Text. Questionnaire**

**Questionnaire used for research “prevalence of anemia and its associated factors among under-five children living in Arba Minch health and demographic surveillance system sites (HDSS)”**

**Part I: Socio-demographic characteristics of under-five children in AM-HDSS sites**

**Instruction**: Fill the information, either thick in the appropriate boxes by using “right” or a word or phrases where required.

| Q. No | Variable | Response | Skip  to |
| --- | --- | --- | --- |
| Q101 | Age of child in months? |  |  |
| Q102 | Sex of child? | 1. Male 2. Female |  |
| Q103 | What is the marital status of mother/care giver? | 1. Married 2. Divorced 3. Widowed 4. Single 5. Other (specify------ |  |
| Q104 | Number of under five children in family | 1. Two 2. >=Three |  |
| Q105 | What is the source of drinking water? | 1. Clean tap water 2. Other source |  |

| Q106 | What is the waste disposal system? | 1. No 2. Pit 3. Burning 4. Open field 5. Garbage can |  |
| --- | --- | --- | --- |
| Q107 | What is the educational level of mother/care giver? | 1. No formal education 2. Primary education 3. Secondary education and above |  |
| Q108 | What is the current occupation of mother/care giver? | 1. House wife 2. Private employee/business 3. Government employee 4. Other. Specify…. |  |
| Q109 | Sex of household head? | 1. Male 2. Female |  |
| Q110 | Number of individuals in the house hold? | 1. Two 2. Three 3. Four 4. Five and above |  |
| Q111 | Place of residence? | 1. Urban 2. Semi-urban 3. Rural |  |

**Part II: Questions to assess child dietary consumption pattern of under-five children in AM-HDSS sites**

**Instruction** –Dear respondent please take few moment to memorize the food and drinks your child ate within the last 1 week. These relate to your daily use of food items and also food consumed out of home, e.g. in a restaurant, bar, at work etc. I will say the food items if you consumed the food type you will tell me how often you ate over the last week.

**If you child eat for instance** ‘Kurkufa’ during four days per week (Monday, Tuesday, Wednesday, and Thursday) you will choose the option that says 2-4 times per week.

| S .no | Food lists | never | 1 day | 2-4 days | 5-6 days | 7 days |
| --- | --- | --- | --- | --- | --- | --- |
| 1 | Moringa/halako |  |  |  |  |  |
| 2 | Kurkuffa/ Ga’aa/ Tsuluka/ Buba/ Eshkirkir |  |  |  |  |  |
| 3 | Fossesie |  |  |  |  |  |
| 4 | Key ye tef Injera |  |  |  |  |  |
| 5 | Nech ye tef injera |  |  |  |  |  |
| 6 | Injera mixed with rice/bokolo |  |  |  |  |  |
| 7 | Macoroni |  |  |  |  |  |
| 8 | Pasta |  |  |  |  |  |
| 9 | Rice |  |  |  |  |  |
| 11 | Bokolo kita |  |  |  |  |  |
| 12 | Sanbusa |  |  |  |  |  |
| 13 | Ambasha(wheat,maize After fermented) |  |  |  |  |  |
| 14 | Bread nech ye sukih/ye furno duket dabo) |  |  |  |  |  |
| 15 | Kinche (oat,Gebs..) |  |  |  |  |  |
| 16 | Nifro (wheat,maize …) |  |  |  |  |  |
| 17 | Nifro (broad bean, kidney been |  |  |  |  |  |
| 18 | Nifro (mixed of wheat and kidney bean, maize ) |  |  |  |  |  |
| 19 | Ye bula genfo |  |  |  |  |  |
| 20 | Ye gebs/teff genfo |  |  |  |  |  |
| 21 | Qolo (wheat, barley) |  |  |  |  |  |

| 22 | Qolo (pea) |  |  |  |  |  |
| --- | --- | --- | --- | --- | --- | --- |
| 23 | Qolo (mixed of wheat and pea) |  |  |  |  |  |
| 24 | Gruel (atmit) |  |  |  |  |  |
| 25 | Ful ye bakela |  |  |  |  |  |
| 26 | Aterkik wet |  |  |  |  |  |
| 27 | Misirkik wet/ split lentil stew |  |  |  |  |  |
| 28 | Yebakela shiro |  |  |  |  |  |
| 29 | Shiro ye aterina shimbira |  |  |  |  |  |
| 30 | Shiro ye atarina bakela |  |  |  |  |  |
| 31 | Mitin shiro |  |  |  |  |  |
| 32 | Sweet potato |  |  |  |  |  |
| 33 | Boyna (Cassava) |  |  |  |  |  |
| 34 | Boye |  |  |  |  |  |
| 35 | Kocho/ Bula |  |  |  |  |  |
| 36 | Carrot with potato stew |  |  |  |  |  |
| 37 | Beetroot stew |  |  |  |  |  |
| 38 | Tikilil gomen |  |  |  |  |  |
| 39 | Tikur gomen (Cabbage) |  |  |  |  |  |
| 40 | Kosta |  |  |  |  |  |
| 41 | Tomato ( silsih,qurt) |  |  |  |  |  |
| 42 | Pumpkin (stew, boiled) |  |  |  |  |  |
| 43 | Fish (fried, stew) |  |  |  |  |  |
| 44 | Yebere Siga (stew, fried, qiqil, Kitfo) |  |  |  |  |  |
| 45 | Yebeg tibs |  |  |  |  |  |
| 46 | Organ meat (heart, kidney and liver) |  |  |  |  |  |
| 47 | Chicken stew |  |  |  |  |  |
| 48 | Egg |  |  |  |  |  |
| 49 | Milk (cow) |  |  |  |  |  |
| 50 | Cheese |  |  |  |  |  |
| 51 | Butter |  |  |  |  |  |
| 52 | Yoghurt |  |  |  |  |  |
| 53 | Honey |  |  |  |  |  |
| 54 | Banana |  |  |  |  |  |
| 55 | Papaya |  |  |  |  |  |
| 56 | Avocado |  |  |  |  |  |
| 57 | Mango |  |  |  |  |  |
| 58 | Orange |  |  |  |  |  |
| 59 | Lemon |  |  |  |  |  |
| 60 | Oil (type of oil)  Plant/palm/ saturated/ yerega |  |  |  |  |  |
| 61 | Jam |  |  |  |  |  |
| 62 | Sugar |  |  |  |  |  |
| 63 | Soft drinks |  |  |  |  |  |
| 64 | Cheka |  |  |  |  |  |
| 65 | Burger |  |  |  |  |  |
| 66 | Pizza |  |  |  |  |  |
| 67 | Cake ( types of cake) |  |  |  |  |  |

**Part III: Health Care and Diseases Characteristics of under-five children living in AM-HDSS sites**

| **No** | **Question** | **Response** | **Skip to** |
| --- | --- | --- | --- |
| Q401 | Have you told by health professional as your child experienced any illness/ infection in the last 2 weeks? | 1. Yes 2. No | If no go to Q403 |
| Q402 | If yes to Q 401, what type of illness did you child experienced? | 1. Diarrhea 2. Malaria 3. Parasitic infection 4. Other specify_________ |  |
| Q403 | History of ANC follow up during this pregnancy | 1. Yes 2. No |  |
| Q404 | Place of delivery for this child | 1. Health care facility 2. Home delivery |  |

**Part IV: Questions to assess the current family wealth (economic condition) of residents in AM-HDSS sites**

**Instruction: Could you kindly tell me if you have the following in your house?**

| **Asset type** | **Response** | |
| --- | --- | --- |
| **Domestic animals** |  |  |
| Ox | No (0) | Yes (1) |
| Cow | No (0) | Yes (1) |
| Calf | No (0) | Yes (1) |
| Sheep | No (0) | Yes (1) |
| Goat | No (0) | Yes (1) |
| Horse | No (0) | Yes (1) |
| Donkey | No (0) | Yes (1) |
| Cock and Hen | No (0) | Yes (1) |
| **Durable assets** |  |  |
| Television | No (0) | Yes (1) |
| Radio | No (0) | Yes (1) |
| Electricity | No (0) | Yes (1) |
| Refrigerator | No (0) | Yes (1) |
| Conventional telephone | No (0) | Yes (1) |
| Mobile phone | No (0) | Yes (1) |
| Car | No (0) | Yes (1) |
| Motorcycle | No (0) | Yes (1) |
| Cycle | No (0) | Yes (1) |
| Cart | No (0) | Yes (1) |
| Gold, money | No (0) | Yes (1) |
| Ownership of owned living house | No (0) | Yes (1) |
| Ownership of agricultural land | No (0) | Yes (1) |
| **Productive assets** |  |  |
| Plough plow | No (0) | Yes (1) |
| Axe | No (0) | Yes (1) |
| Hoe | No (0) | Yes (1) |
| Shovel | No (0) | Yes (1) |
| Sickle | No (0) | Yes (1) |
| Modern beehive | No (0) | Yes (1) |
| Traditional beehive | No (0) | Yes (1) |
| **Housing characteristics** |  |  |
| Indoor plumping/ pipe water | No (0) | Yes (1) |
| Type of flooring | Earth/dung (0) | Cement/raw wood (1) |
| **Other household materials** |  |  |
| Bed | No (0) | Yes (1) |
| Table | No (0) | Yes (1) |
| Chair | No (0) | Yes (1) |
| Stove | No (0) | Yes (1) |

**Part V: Questions to assess household food security condition (HFIAS)**

| **Q. No** | **Questions** | **Response options (encircle one)** | | | **skip** |
| --- | --- | --- | --- | --- | --- |
| 601 | In the past four weeks, did you worry that your household would not have enough food? | | 0 = No  1=Yes | | If 0, Q 602 |
| 601.a | How often did this happen? | 1 = Rarely (1X or 2X in the past four weeks)  2 = Sometimes (3x to 10x in the past four weeks)  3 = Often (>10x in the past four weeks) | | |  |
| 602 | In the past four weeks, were you or any household member not able to eat the kinds of foods you preferred because of a lack of resources? | | 0 = No  1=Yes | | If 0, to Q 603 |
| 602.a | How often did this happen? | 1 = Rarely (1X or 2X in the past 4 weeks)  2 = Sometimes (3x to 10x in the past four weeks)  3 = Often (>10x in the past four weeks) | | |  |
| 603 | In the past four weeks, did you or any household member have to eat a limited variety of foods due to a lack of resources? | | 0 = No  1=Yes | | If 0, to Q 604 |
| 603.a | How often did this happen? | 1 = Rarely (1X or 2X in the past four weeks)  2 = Sometimes (3x to 10x in the past four weeks)  3 = Often (>10x in the past four weeks) | | |  |
| 604 | In the past four weeks, did you or any household member have to eat some foods that you really did not want to eat because of a lack of resources to obtain other types of food? | | 0 = No  1=Yes | | If 0, to Q 605 |
| 604.a | How often did this happen? | 1 = Rarely (1X or 2X in the past four weeks)  2 = Sometimes (3x to 10x in the past four weeks)  3 = Often (>10x in the past four weeks) | | |  |
| 605 | In the past four weeks, did you or any household member have to eat a smaller meal than you felt you needed because there was not enough food? | | 0 = No  1=Yes | | If 0, to Q 606 |
| 605.a | How often did this happen? | 1 = Rarely (1X or 2X in the past four weeks)  2 = Sometimes (3x to 10x in the past four weeks)  3 = Often (>10x in the past four weeks) | | |  |
| 606 | In the past four weeks, did you or any other household member have to eat fewer meals in a day because there was not enough food? | | 0 = No  1=Yes | | If 0, to Q 607 |
| 606.a | How often did this happen? | 1 = Rarely (1X or 2X in the past four weeks)  2 = Sometimes (3x to 10x in the past four weeks)  3 = Often (>10x in the past four weeks) | | |  |
| 607 | In the past four weeks, was there ever no food to eat of any kind in your household because of lack of resources to get food? | | 0 = No  1=Yes | | If 0, to Q 608 |
| 607.a | How often did this happen? | 1 = Rarely (1X or 2X in the past four weeks)  2 = Sometimes (3x to 10x in the past four weeks)  3 = Often (>10x in the past four weeks) | | |  |
| 608 | In the past four weeks, did you or any household member go to sleep at night hungry because there was not enough food? | | 0 = No  1=Yes | | If 0, to Q 609 |
| 608.a | How often did this happen? | 1 = Rarely (1X or 2X in the past four weeks)  2 = Sometimes (3x to 10x in the past four weeks)  3 = Often (>10x in the past four weeks) | | |  |
| 609 | In the past four weeks, did you or any household member go a whole day and night without eating anything because there was not enough food | | | 0 = No (Questionnaire finished)  1=Yes | If 0, finish the questions |
| 609.a | How often did this happen? | 1 = Rarely (1X or 2X in the past four weeks)  2 = Sometimes (3x to 10x in the past four weeks)  3 = Often (>10x in the past four weeks) | | |  |
