# Supplementary material for: Prevalence of anemia and its associated factors among under-five children living in Arba Minch Health and Demographic Surveillance System Sites (HDSS), Southern Ethiopia
Source: PLOS Glob Public Health. 2024 Nov 5;4(11):e0003830. doi: 10.1371/journal.pgph.0003830 (PMC11537392; doi:10.1371/journal.pgph.0003830)
Supplement: Attachment — Submitted filename: Response to Reviewers.docx [file pgph.0003830.s002.docx]

**From: PLOS Global Public Health**

Date: Fri, Aug 23, 2024 at 9:34 PM

Subject: PLOS Global Public Health Decision: Revision required [PGPH-D-24-01672] - [EMID:51e10d88c971d4ba]

To: Samuel Alemu Bamboro <bizuneshhaile23@gmail.com>

PGPH-D-24-01672

Prevalence of anemia and its associated factors among under-five children living in Arba Minch Health and Demographic Surveillance System Sites (HDSS), Southern Ethiopia

PLOS Global Public Health

**Dear Dr. Bamboro,**

Thank you for submitting your manuscript to PLOS Global Public Health. After careful consideration, we feel that it has merit but does not fully meet PLOS Global Public Health’s publication criteria as it currently stands. Therefore, we invite you to submit a revised version of the manuscript that addresses the points raised during the review process.

Please submit your revised manuscript by Oct 07 2024 11:59PM. If you will need more time than this to complete your revisions, please reply to this message or contact the journal office at globalpubhealth@plos.org. When you're ready to submit your revision, log on to https://www.editorialmanager.com/pgph/ and select the 'Submissions Needing Revision' folder to locate your manuscript file.

Please include the following items when submitting your revised manuscript:

A rebuttal letter that responds to each point raised by the editor and reviewer(s). You should upload this letter as a separate file labeled 'Response to Reviewers'.

A marked-up copy of your manuscript that highlights changes made to the original version. You should upload this as a separate file labeled 'Revised Manuscript with Track Changes'.

An unmarked version of your revised paper without tracked changes. You should upload this as a separate file labeled 'Manuscript'.

Guidelines for resubmitting your figure files are available below the reviewer comments at the end of this letter.

We look forward to receiving your revised manuscript.

Kind regards,

Kamala Thriemer

Academic Editor

PLOS Global Public Health

**Dear Editor Kamala Thriemer and Reviewers,**

We sincerely appreciate your thoughtful and constructive feedback, which has greatly contributed to improving our manuscript. Below, we have provided detailed responses to the reviewers' comments, and all revisions made to the manuscript are clearly marked using track changes.

We hope that the revisions align with your expectations, and we look forward to your favorable consideration for publication.

**Sincerely,**

**Samuel Alemu Bamboro**

**Comments to the Author**

1. Does this manuscript meet PLOS Global Public Health’s [publication criteria](https://c05y1x9s.r.us-east-2.awstrack.me/L0/https:%2F%2Fjournals.plos.org%2Fglobalpublichealth%2Fs%2Fcriteria-for-publication/1/010f01918084c8f5-e657882b-539a-4414-a798-08f9def5020e-000000/xBTkMkTg56RVFTr2Dh2POo_5CHw=173)? Is the manuscript technically sound, and do the data support the conclusions? The manuscript must describe methodologically and ethically rigorous research with conclusions that are appropriately drawn based on the data presented.

Reviewer #1: Yes

Reviewer #2: Partly

2. Has the statistical analysis been performed appropriately and rigorously?

Reviewer #1: Yes

Reviewer #2: Yes

3. Have the authors made all data underlying the findings in their manuscript fully available (please refer to the Data Availability Statement at the start of the manuscript PDF file)?

The [PLOS Data policy](https://c05y1x9s.r.us-east-2.awstrack.me/L0/https:%2F%2Fjournals.plos.org%2Fglobalpublichealth%2Fs%2Fdata-availability/1/010f01918084c8f5-e657882b-539a-4414-a798-08f9def5020e-000000/3aen-1hN4vWBBMe8FDWSHENuwts=173) requires authors to make all data underlying the findings described in their manuscript fully available without restriction, with rare exception. The data should be provided as part of the manuscript or its supporting information, or deposited to a public repository. For example, in addition to summary statistics, the data points behind means, medians and variance measures should be available. If there are restrictions on publicly sharing data—e.g. participant privacy or use of data from a third party—those must be specified.

Reviewer #1: Yes

Reviewer #2: No

4. Is the manuscript presented in an intelligible fashion and written in standard English?

PLOS Global Public Health does not copyedit accepted manuscripts, so the language in submitted articles must be clear, correct, and unambiguous. Any typographical or grammatical errors should be corrected at revision, so please note any specific errors here.

Reviewer #1: Yes

Reviewer #2: No

5. **Review Comments to the Author**

Please use the space provided to explain your answers to the questions above. You may also include additional comments for the author, including concerns about dual publication, research ethics, or publication ethics. (Please upload your review as an attachment if it exceeds 20,000 characters)

**Reviewer #1:**

This paper presents the results of a cross-sectional survey of anaemia prevalence and associated socio-economic and demographic risk factors in children under 5 years residing in **one of 9 kebeles in Arba Minch District in Ethiopia**. The study is reported clearly. The sampling frame was appropriate. Data collection procedures were robust (including forward and back translation between English and Amharic) and the analysis methods are satisfactory. The survey showed that anaemia (haemoglobin <11g/dL) was present in 35.3% of children and was negatively associated with age and recent consumption of fruits and vegetables and positively associated with increasing food insecurity. Although valid, these results are not surprising or particularly novel. The study would have been more useful if the authors had also assessed the prevalence of several other very important risk factors such as malaria, helminth infection, specific nutritional deficiencies and red cell and haemoglobin disorders.

**Response:** We thank you for accepting and reviewing the manuscript, along with your valuable comments and suggestions. Eight out of the nine kebeles in the Arba Minch HDSS were included in this study, as indicated in the "Materials and Methods" section under the "Study Area" subsection. One Kebele was excluded due to security concerns during the study period.

Regarding the inclusion of additional variables, the reviewer is correct. However, we only included the variable "history of recent illness," as noted in the "Feeding Practice and Healthcare-Related Factors of the Child and Households" section of the results. During the analysis, this variable was excluded from the multivariate analysis, but we did assess its potential effect by adding it while conducting preliminary tests with the data.

**My suggestions to the authors are all minor:**

1. It is important that the authors state what the definitions of anaemia, moderate and severe anaemia are in the abstract

**Response**: The definition and classifications of anemia is added in the “methods” section of the abstract.

2. Throughout the paper, including the abstract, the authors should specify the directions of the associations found between anaemia and the socioeconomic and demographic risk factors – eg “a negative association with advancing age”

**Response**: The suggestion is included in the manuscript.

3. In the results section, age should be presented with range as well as standard deviation

**Response**: We included in table 1 of the result part.

4. In table 1, for consistency, suggest presenting percentages to the same number of decimal places even though it is clear that these are whole numbers

**Response**: Corrected in the manuscript

**Reviewer #2:**

Page 2 (Introduction): In the Abstract (conclusions), the phrase “Improvement of wealth status” seems inappropriate as the study has tackled wealth status, but not improvement of wealth status.

**Response:** **Thank you for accepting our manuscript for review and for your valuable comments.** We have made the necessary corrections in the manuscript as per your suggestions.

The given definition of anemia mentions decreased capacity to transport oxygen. Adding a qualifying phrase like “blood disorders” “not enough RBCs”, etc. might help.

**Response**: We incorporated the additional suggestions provided in the manuscript.

Page 3 (Introduction), there is attempt to show the link between anemia and several factors such as age, sex, residency, initiation of complementary food, etc. Due to the fact that the citation was not contextual, the statement can be a misleading interpretation of the cited literature. On their own right, age, sex or residency cannot be linked to anemia. The preceding statements have already shown that globally a good percentage of children under 5 are anemic. Similarly, initiation of complementary food is an intervention to deal with under-nutrition, yet also linked to anemia. In this scenario, one would ask whether it is under-nutrition or initiation of complementary food that should be linked to anemia? In other words, the statement does not make it exclusive how age, sex & residency are linked with anemia. Please contextualize. “Studies also indicated age and sex of the child, residency, under nutrition (stunting, wasting and thinness), early initiation of complementary food, maternal health and education status, and low socioeconomic status were linked with anemia (Belachew & Tewabe, 2020; Cardoso et al., 2012; Endris et al., 2022; Gebrie & Alebel, 2020; Ngesa & Mwambi, 2014)”.

**Response:** We re-wrote the sentences accordingly.

**Results**

It seems the data on hemoglobin levels has not been interrogated very well. No table is given, which could be useful. A simple categorization of children by age, sex, wealth status, infection status, maternal health status, etc, would probably give additional information. It would have also helped if data on height and body weight were available and analyzed so that nutritional status (including stunting, wasting, thinness) could be assessed. I would think this is a missed opportunity.

**Response**: We make changes on Table 1 by providing additional information for those with anemia and normal with characteristics of the participants.

Page 8: How was it possible to verify if children had intestinal parasites or malaria? Was this documented? I am referring to the statement “Among those, 13(31%), 15(35.7%), 4(9.5%), and 37(88.1%) children had a history of malaria, diarrhea, intestinal parasites, and fever respectively.”

**Response**: We considered two step question. The first question was about the child’s experience of any illnesses in the preceding two weeks of data collection period. The second, which was described in the finding (and indicated above) was asked if they know their child’s cause of the sickness. To remove the confusion for the readers, we added additional explanations in the method part “Data collection procedure and instruments” of the manuscript.

Page 10: As mentioned in my comments of the abstract section, **food security status of families was assessed but not the increase in wealth score**. Perhaps replacing the words “increasing” or “decreasing” may improve the sentence. See the quote below.

- “The likelihood of anemia decreased by 0.95 (0.93, 0.97) with an increase in the age of the child in a month.
- Similarly, a unit increase in wealth score decreased anemia prevalence with the odds of 0.86 (0.75, 0.99) among children (Table.4).”

**Response: Thank you for the suggestion.** Both the wealth score and food security status of the household were assessed using different tools. For the wealth score, household assets and characteristics were considered and estimated using Principal Component Analysis (PCA), while food security status was collected using the Household Food Insecurity Access Scale (HFIAS) developed by FANTA. We also checked for multicollinearity of the variables using Variance Inflation Factor (VIF) and tolerance, and the VIF values were below 10, indicating no multicollinearity concerns. We have considered and addressed the terms and points indicated.

**Discussion**

1) There is a sudden mention of milk consumption in page 11. The logistic regression did not include data on frequency of food item consumption. This would have made it easier to understand why the authors suddenly mentioned milk consumption in page 11, in which it was indicated the study did not confirm the impact of milk confirmation on children’s iron status.

**Response: In the Methods section, under the "Data Processing and Analysis" subsection,** in addition to using the FFQ to determine the dietary diversity of the child, we also considered meat, fish, and poultry; milk and milk products; and fruit and vegetable consumption as additional variables. However, we initially did not include these variables in the Introduction. We have now added a statement in the Introduction to indicate their relationship with anemia.

2) In Page 12 (1st paragraph) there is mention of contributions of infection/inflammation as being significant. It is unclear if this was a general statement or a reference to the study findings. As I understand it, neither malaria nor intestinal parasites were investigated by way of laboratory testing. By the same token, there were no measurements of iron, folate, vitamin B12 and vitamin A. The authors would need to convey their message in a different way to avoid confusions.

**Response**: We have made the necessary corrections in the manuscript as per your suggestions.

3) Overall, it seemed much effort was not exerted to collect as much relevant data as possible. The discussion is short. Either data is not available or not explored adequately.

**Response:** **We thank the reviewer for their comments and suggestions.** While there are variables that we did not include due to various reasons, such as parasite investigation and biomarker checkups, we aimed to capture the most important factors as covariates for this study, as detailed in the Methods and Results sections. During the analysis, we found that most of the variables were not associated with the outcome.

**English corrections (examples)**

There are a lot of flaws in the use of English language (grammar, syntax, choice of words, etc..) that need to be addressed. Just some examples: In the abstract, the phrase “were participated”, perhaps should just be “participated”

**Response**: Corrections were made in the document.

In Page 4, first sentence mentions “….. there is a huge difference between anemia prevalence and factors associated with anemia among studies conducted in different places (22)”. Here the comparison is **about study sites (or the different studies**), but the sentence seems to compare anemia prevalence with factors associated. This just needs differentiating the subjects of the sentence.

**Response**: Corrected in the manuscript.

Page 4, last sentence of the introduction begins with “So, we would like to see the situation…. “. May be use phrases like “We sought to …….”.

**Response:** Corrected in the manuscript.

Page 4 (last paragraph): Exclusion of severely ill children did make sense to a certain extent, but the impact is less clear as there are no biological measurements.

**Response**: Thank you for your question. We have explained the following concept in the manuscript: as part of our exclusion criteria, we considered the severity of the illness and admission to a health facility, as it was challenging to reach these individuals during data collection. The basis for determining the severity of the illness was admission to a health facility.

Page 5: Verb is missing in the sentence “The frequencies of consumption of over 67 locally available food ingredients for a week before the data was collected for the assessment of dietary diversity score.”

**Response**: Corrected in the manuscript.

Page 6: the last phrase “was used to collect the data” in the sentence “The questionnaire was uploaded on KOBO collect survey, a mobile application that allows for collecting data using mobile devices, was used to collect the data.“ is repetition. It can be deleted.

**Response**: Corrected in the manuscript.

Page 6: The paragraph on data quality assurance and control seems to require re-writing since it has repetitive phrases and statements. See below in the qoute:

- “The questionnaire was prepared in English, translated into the local language (Amharic) and then translated back into English to check consistency. Training was given to the data collectors on the study objective, requesting consent, and data collection techniques for the data collectors. Then the data was collected using KOBO collect. One of the investigators (SAB) closely monitored the data collection process with the server and onsite supervision. Measurements were taken by following the recommended standards of the manufacturer. “

**Response**: Corrected in the manuscript.

Page 6: The statement “Data was collected by using the KOBO collect” repeats several times.

**Response**: Corrected in the manuscript.

Page 7: The phrase “there was no correlation between this independent variable” needs be explicit. It is unclear which variables are referred to. Furthermore, some sections of the same paragraph, unless it is re-written in a different way, seem to be a description of results, not expected in the M&M section. See the paragraph quoted below.

“Multicollinearity was assessed by examining the Variance Inflation Factor (VIF) and tolerance, which indicates that there was no correlation between this independent variable. The final model was tested for its fitness by Hosmer and Lemeshow p-value and the p-value was 0.879, which indicates the good fitness of the model.”

**Response**: Corrected in the manuscript.

Page 11 (Discussion), please correct the phrase “in younger aged. ..”

**Response**: Corrected in the manuscript.

Page 11 (Discussion), please correct the English in the phrase “For the most of the ….”.

**Response**: Corrected in the manuscript.

Page 11 (Discussion), please correct the English in the phrase “for access adequate….”

**Response**: Corrected in the manuscript.

Page 12 (1st paragraph): please correct the English in the phrase “non-nutrition related factors also contribution ……….”.

**Response**: Corrected in the manuscript.

**References**

Journal names are given either in full designations or in abbreviated form. Be consistent.

**Response**: Checked and corrected according to your comment.

6. PLOS authors have the option to publish the peer review history of their article ([what does this mean?](https://c05y1x9s.r.us-east-2.awstrack.me/L0/https:%2F%2Fjournals.plos.org%2Fglobalpublichealth%2Fs%2Feditorial-and-peer-review-process%23loc-peer-review-history/1/010f01918084c8f5-e657882b-539a-4414-a798-08f9def5020e-000000/aHjWGs1MYH6YDGrY05dVIb5GIcY=173)). If published, this will include your full peer review and any attached files.

**Do you want your identity to be public for this peer review?** If you choose “no”, your identity will remain anonymous but your review may still be made public.

For information about this choice, including consent withdrawal, please see our [Privacy Policy](https://c05y1x9s.r.us-east-2.awstrack.me/L0/https:%2F%2Fwww.plos.org%2Fprivacy-policy/1/010f01918084c8f5-e657882b-539a-4414-a798-08f9def5020e-000000/htf_QueGFOdnC91gGxvQq4j5164=173).

Reviewer #1: No

Reviewer #2: No
